# 1-Meth­oxy-4-({[(4-meth­oxy­phen­yl)­sulfan­yl](phen­yl)meth­yl}sulfan­yl)benzene

**DOI:** 10.1107/S1600536812006320

**Published:** 2012-02-17

**Authors:** Hongqi Li, G. Ramachandran, M. Sathishkumar, K. Sathiyanarayanan, R. S. Rathore

**Affiliations:** a Key Laboratory of Science and Technology of Eco-Textiles, Ministry of Education, College of Chemistry, Chemical Engineering and Biotechnology, Donghua University, Shanghai 201620, People’s Republic of China; b Chemistry Division, School of Advanced Sciences, VIT University, Vellore 632 014, India; c Bioinformatics Infrastructure Facility, Department of Biotechnology, School of Life Sciences, University of Hyderabad, Hyderabad 500 046, India

## Abstract

The title compound, C_21_H_20_O_2_S_2_, forms a propeller-shaped structure with the tetra­hedral C atom as the central hub and meth­oxy­benzene and phenyl residues as radiating blades. Short C—H⋯π contacts are observed.

## Related literature

For related structures, see: Farrugia *et al.* (2000 ▶). For details on the Cambridge Structural Database, see: Allen (2002 ▶).
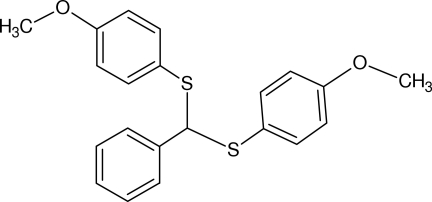


## Experimental

### 

#### Crystal data

C_21_H_20_O_2_S_2_
*M* *_r_* = 368.49
Monoclinic, 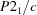

*a* = 21.1506 (15) Å
*b* = 5.6114 (3) Å
*c* = 17.1219 (11) Å
β = 110.336 (2)°
*V* = 1905.4 (2) Å^3^
*Z* = 4
Mo *K*α radiation
μ = 0.29 mm^−1^
*T* = 298 K
0.42 × 0.20 × 0.18 mm

#### Data collection

Bruker APEXII CCD area-detector diffractometer
Absorption correction: multi-scan (*SADABS*; Bruker, 2004 ▶) *T*_min_ = 0.888, *T*_max_ = 0.950
12756 measured reflections
4154 independent reflections
2725 reflections with *I* > 2σ(*I*)
*R*_int_ = 0.024

#### Refinement

*R*[*F* ^2^ > 2σ(*F* ^2^)] = 0.043
*wR*(*F* ^2^) = 0.128
*S* = 1.02
4154 reflections
228 parameters
H-atom parameters constrained
Δρ_max_ = 0.51 e Å^−3^
Δρ_min_ = −0.27 e Å^−3^

### 

Data collection: *APEX2* (Bruker, 2004 ▶); cell refinement: *APEX2*; data reduction: *SAINT-Plus* (Bruker, 2004 ▶); program(s) used to solve structure: *SHELXS97* (Sheldrick, 2008 ▶); program(s) used to refine structure: *SHELXL97* (Sheldrick, 2008 ▶); molecular graphics: *ORTEP-3* (Farrugia, 1997 ▶); software used to prepare material for publication: *SHELXL97* and *PLATON* (Spek, 2009 ▶).

## Supplementary Material

Crystal structure: contains datablock(s) global, I. DOI: 10.1107/S1600536812006320/tk5059sup1.cif

Structure factors: contains datablock(s) I. DOI: 10.1107/S1600536812006320/tk5059Isup2.hkl

Supplementary material file. DOI: 10.1107/S1600536812006320/tk5059Isup3.cml

Additional supplementary materials:  crystallographic information; 3D view; checkCIF report

## Figures and Tables

**Table 1:** Hydrogen-bond geometry (Å, °) *Cg*1 and *Cg*2 are the centroids of the C1–C6 and C8–C13 rings, respectively.

*D*—H⋯*A*	*D*—H	H⋯*A*	*D*⋯*A*	*D*—H⋯*A*
C4—H4⋯*Cg*2^i^	0.93	2.99	3.713 (3)	136
C10—H10⋯*Cg*2^ii^	0.93	2.96	3.610 (2)	128
C21—H21*B*⋯*Cg*1^iii^	0.96	2.98	3.914 (5)	165

Symmetry codes: (i) 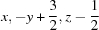; (ii) 
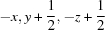
; (iii) 
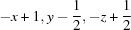
.

**Fig. 1.**
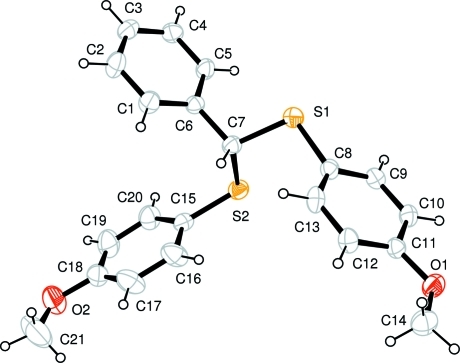
A view of the title compound with non-H atoms shown with 30% probability ellipsoids.

## supplementary crystallographic information

### Comment

A search in Cambridge Structural Database (CCDC; version 5.31; Allen, 2002) revealed four similar crystal structures (Farrugia *et al.*, 2000). The title compound, Fig. 1, forms a propeller-shaped structure with the tetrahedral carbon of the phenylmethyl moiety as the central hub and methoxybenzene and phenyl moieties as radiating blades. Short C—H···π contacts stabilize the structure, Table 1.

### Experimental

In a dry 100 ml Erlenmeyer flask were placed benzaldehyde (10 mmol), 4-methoxythiophenol (10 mmol), iodine (15 mol %) and dichloromethane (DCM; 15 ml). The reaction mixture was stirred at room temperature for an hour. The reaction was monitored by TLC and after the completion of reaction the iodine utilized was removed from the product by treating it with aqueous sodium thiosulfate solution. The final product was extracted into DCM (2 *x* 20 ml). The crude reaction mixture was purified by column chromatography on silica gel using ethyl acetate/hexane as the eluents. Final yields: 93%; *M*.pt: 322 (1) °K. Suitable single crystals of the title compound were grown from its ethanol/tetrahydrofuran mixture (1:1).

### Refinement

H atoms were placed in their geometrically expected positions and refined in the riding-model approximation [C*sp*^2^—H = 0.93 Å, C(methine)—H = 0.98 Å and C(methyl)—H = 0.96 Å, and with *U*_iso_(H) = 1.2*U*_eq_(parent) or 1.5*U*_eq_(C_methyl_)]. The torsion angles for the methyl group H atoms were set with reference to a local difference Fourier calculation. The reflection (100), truncated by the beam stop, was omitted during the final cycles of refinement.

### Crystal data

**Table:**

C_21_H_20_O_2_S_2_	*F*(000) = 776
*M**_r_* = 368.49	*D*_x_ = 1.285 Mg m^−^^3^
Monoclinic, *P*2_1_/*c*	Melting point: 322(1) K
Hall symbol: -P 2ybc	Mo *K*α radiation, λ = 0.71073 Å
*a* = 21.1506 (15) Å	Cell parameters from 4040 reflections
*b* = 5.6114 (3) Å	θ = 2.5–25.1°
*c* = 17.1219 (11) Å	µ = 0.29 mm^−^^1^
β = 110.336 (2)°	*T* = 298 K
*V* = 1905.4 (2) Å^3^	Rectangular, colourless
*Z* = 4	0.42 × 0.20 × 0.18 mm

### Data collection

**Table:**

Bruker APEXII CCD area-detector diffractometer	4154 independent reflections
Radiation source: fine-focus sealed tube	2725 reflections with *I* > 2σ(*I*)
Graphite monochromator	*R*_int_ = 0.024
φ and ω scans	θ_max_ = 27.3°, θ_min_ = 2.1°
Absorption correction: multi-scan (*SADABS*; Bruker, 2004)	*h* = −27→26
*T*_min_ = 0.888, *T*_max_ = 0.950	*k* = −6→7
12756 measured reflections	*l* = −21→21

### Refinement

**Table:**

Refinement on *F*^2^	Primary atom site location: structure-invariant direct methods
Least-squares matrix: full	Secondary atom site location: difference Fourier map
*R*[*F*^2^ > 2σ(*F*^2^)] = 0.043	Hydrogen site location: inferred from neighbouring sites
*wR*(*F*^2^) = 0.128	H-atom parameters constrained
*S* = 1.02	*w* = 1/[σ^2^(*F*_o_^2^) + (0.0584*P*)^2^ + 0.5757*P*] where *P* = (*F*_o_^2^ + 2*F*_c_^2^)/3
4154 reflections	(Δ/σ)_max_ < 0.001
228 parameters	Δρ_max_ = 0.51 e Å^−^^3^
0 restraints	Δρ_min_ = −0.27 e Å^−^^3^

### Special details

**Table:**

Geometry. All e.s.d.'s (except the e.s.d. in the dihedral angle between two l.s. planes) are estimated using the full covariance matrix. The cell e.s.d.'s are taken into account individually in the estimation of e.s.d.'s in distances, angles and torsion angles; correlations between e.s.d.'s in cell parameters are only used when they are defined by crystal symmetry. An approximate (isotropic) treatment of cell e.s.d.'s is used for estimating e.s.d.'s involving l.s. planes.
Refinement. Refinement of *F*^2^ against ALL reflections. The weighted *R*-factor *wR* and goodness of fit *S* are based on *F*^2^, conventional *R*-factors *R* are based on *F*, with *F* set to zero for negative *F*^2^. The threshold expression of *F*^2^ > σ(*F*^2^) is used only for calculating *R*-factors(gt) *etc*. and is not relevant to the choice of reflections for refinement. *R*-factors based on *F*^2^ are statistically about twice as large as those based on *F*, and *R*- factors based on ALL data will be even larger.

### Fractional atomic coordinates and isotropic or equivalent isotropic displacement parameters (Å^2^)

**Table:**

	*x*	*y*	*z*	*U*_iso_*/*U*_eq_	
C1	0.28208 (12)	0.3397 (4)	0.08333 (15)	0.0582 (6)	
H1	0.2956	0.2300	0.1267	0.070*	
C2	0.30050 (14)	0.3069 (5)	0.01429 (17)	0.0682 (7)	
H2	0.3269	0.1764	0.0118	0.082*	
C3	0.28023 (12)	0.4651 (5)	−0.05079 (14)	0.0582 (6)	
H3	0.2923	0.4412	−0.0975	0.070*	
C4	0.24238 (12)	0.6575 (4)	−0.04643 (14)	0.0579 (6)	
H4	0.2286	0.7656	−0.0903	0.069*	
C5	0.22434 (11)	0.6929 (4)	0.02289 (14)	0.0553 (6)	
H5	0.1988	0.8255	0.0255	0.066*	
C6	0.24388 (10)	0.5330 (4)	0.08852 (12)	0.0437 (5)	
C7	0.22303 (10)	0.5625 (4)	0.16395 (13)	0.0472 (5)	
H7	0.2331	0.4144	0.1963	0.057*	
C8	0.11543 (10)	0.5823 (4)	0.22356 (13)	0.0465 (5)	
C9	0.07731 (10)	0.7557 (4)	0.24299 (13)	0.0498 (5)	
H9	0.0650	0.8914	0.2099	0.060*	
C10	0.05727 (10)	0.7304 (4)	0.31070 (13)	0.0497 (5)	
H10	0.0306	0.8472	0.3223	0.060*	
C11	0.07636 (10)	0.5324 (4)	0.36186 (13)	0.0463 (5)	
C12	0.11566 (11)	0.3596 (4)	0.34425 (15)	0.0534 (6)	
H12	0.1297	0.2275	0.3788	0.064*	
C13	0.13406 (11)	0.3849 (4)	0.27413 (15)	0.0561 (6)	
H13	0.1594	0.2660	0.2612	0.067*	
C14	0.06989 (14)	0.3243 (5)	0.48056 (16)	0.0749 (8)	
H14A	0.0515	0.1833	0.4491	0.112*	
H14B	0.0514	0.3439	0.5240	0.112*	
H14C	0.1180	0.3095	0.5047	0.112*	
C15	0.34680 (11)	0.6870 (4)	0.27962 (13)	0.0504 (5)	
C16	0.35770 (14)	0.5044 (6)	0.33572 (17)	0.0822 (9)	
H16	0.3216	0.4438	0.3486	0.099*	
C17	0.42130 (16)	0.4077 (6)	0.37378 (19)	0.0917 (10)	
H17	0.4276	0.2804	0.4105	0.110*	
C18	0.47508 (14)	0.5028 (6)	0.35644 (17)	0.0741 (8)	
C19	0.46437 (15)	0.6859 (6)	0.3018 (2)	0.0877 (9)	
H19	0.5007	0.7511	0.2906	0.105*	
C20	0.40113 (13)	0.7771 (5)	0.26288 (17)	0.0718 (7)	
H20	0.3949	0.9008	0.2248	0.086*	
C21	0.5537 (2)	0.2212 (8)	0.4413 (3)	0.1463 (19)	
H21A	0.5398	0.2463	0.4884	0.220*	
H21B	0.6011	0.1866	0.4603	0.220*	
H21C	0.5291	0.0896	0.4090	0.220*	
O1	0.05333 (8)	0.5258 (3)	0.42700 (10)	0.0633 (4)	
O2	0.54066 (11)	0.4281 (5)	0.39166 (15)	0.1103 (8)	
S1	0.13305 (3)	0.61593 (13)	0.12994 (4)	0.0614 (2)	
S2	0.26527 (3)	0.80951 (11)	0.23220 (3)	0.05424 (19)	

### Atomic displacement parameters (Å^2^)

**Table:**

	*U*^11^	*U*^22^	*U*^33^	*U*^12^	*U*^13^	*U*^23^
C1	0.0697 (15)	0.0523 (15)	0.0573 (14)	0.0096 (11)	0.0281 (12)	0.0104 (11)
C2	0.0824 (18)	0.0560 (16)	0.0804 (18)	0.0158 (13)	0.0463 (15)	0.0015 (14)
C3	0.0652 (15)	0.0657 (16)	0.0505 (13)	−0.0064 (12)	0.0287 (11)	−0.0081 (12)
C4	0.0617 (14)	0.0656 (16)	0.0474 (13)	0.0037 (12)	0.0203 (11)	0.0117 (11)
C5	0.0600 (14)	0.0544 (14)	0.0561 (13)	0.0129 (11)	0.0262 (11)	0.0068 (11)
C6	0.0443 (11)	0.0451 (12)	0.0425 (11)	−0.0031 (9)	0.0160 (9)	−0.0024 (9)
C7	0.0485 (11)	0.0502 (13)	0.0447 (11)	−0.0023 (10)	0.0183 (9)	−0.0008 (10)
C8	0.0387 (10)	0.0504 (13)	0.0508 (12)	−0.0041 (9)	0.0162 (9)	−0.0030 (10)
C9	0.0435 (11)	0.0502 (14)	0.0541 (13)	0.0018 (10)	0.0149 (9)	0.0050 (10)
C10	0.0452 (11)	0.0468 (13)	0.0590 (13)	0.0059 (9)	0.0206 (10)	−0.0026 (11)
C11	0.0388 (11)	0.0511 (13)	0.0502 (12)	−0.0024 (9)	0.0169 (9)	−0.0016 (10)
C12	0.0497 (12)	0.0449 (13)	0.0688 (15)	0.0040 (10)	0.0250 (11)	0.0089 (11)
C13	0.0490 (12)	0.0469 (14)	0.0801 (16)	0.0039 (10)	0.0322 (12)	−0.0059 (12)
C14	0.0818 (19)	0.082 (2)	0.0701 (17)	0.0028 (15)	0.0380 (15)	0.0216 (15)
C15	0.0551 (13)	0.0552 (14)	0.0423 (11)	−0.0060 (10)	0.0186 (9)	−0.0050 (10)
C16	0.0661 (17)	0.102 (2)	0.0743 (17)	−0.0057 (16)	0.0187 (14)	0.0297 (17)
C17	0.086 (2)	0.096 (2)	0.077 (2)	0.0070 (18)	0.0083 (16)	0.0303 (17)
C18	0.0609 (16)	0.088 (2)	0.0660 (16)	0.0093 (15)	0.0134 (13)	−0.0180 (15)
C19	0.0616 (17)	0.101 (3)	0.109 (2)	−0.0023 (16)	0.0406 (17)	0.006 (2)
C20	0.0687 (17)	0.0734 (18)	0.0814 (18)	−0.0002 (14)	0.0362 (14)	0.0113 (15)
C21	0.115 (3)	0.132 (4)	0.138 (4)	0.051 (3)	−0.025 (3)	−0.013 (3)
O1	0.0678 (10)	0.0719 (12)	0.0588 (9)	0.0083 (9)	0.0329 (8)	0.0075 (9)
O2	0.0726 (14)	0.136 (2)	0.1071 (17)	0.0291 (14)	0.0116 (12)	−0.0111 (16)
S1	0.0485 (3)	0.0866 (5)	0.0493 (3)	0.0027 (3)	0.0174 (2)	0.0004 (3)
S2	0.0579 (3)	0.0549 (4)	0.0504 (3)	−0.0013 (3)	0.0195 (3)	−0.0066 (3)

### Geometric parameters (Å, º)

**Table:**

C1—C6	1.374 (3)	C12—C13	1.392 (3)
C1—C2	1.379 (3)	C12—H12	0.9300
C1—H1	0.9300	C13—H13	0.9300
C2—C3	1.372 (3)	C14—O1	1.421 (3)
C2—H2	0.9300	C14—H14A	0.9600
C3—C4	1.361 (3)	C14—H14B	0.9600
C3—H3	0.9300	C14—H14C	0.9600
C4—C5	1.381 (3)	C15—C16	1.368 (3)
C4—H4	0.9300	C15—C20	1.374 (3)
C5—C6	1.384 (3)	C15—S2	1.770 (2)
C5—H5	0.9300	C16—C17	1.385 (4)
C6—C7	1.512 (3)	C16—H16	0.9300
C7—S1	1.811 (2)	C17—C18	1.379 (4)
C7—S2	1.833 (2)	C17—H17	0.9300
C7—H7	0.9800	C18—C19	1.354 (4)
C8—C9	1.376 (3)	C18—O2	1.372 (3)
C8—C13	1.377 (3)	C19—C20	1.370 (4)
C8—S1	1.778 (2)	C19—H19	0.9300
C9—C10	1.373 (3)	C20—H20	0.9300
C9—H9	0.9300	C21—O2	1.408 (5)
C10—C11	1.385 (3)	C21—H21A	0.9600
C10—H10	0.9300	C21—H21B	0.9600
C11—O1	1.364 (2)	C21—H21C	0.9600
C11—C12	1.377 (3)		
			
C6—C1—C2	120.6 (2)	C13—C12—H12	120.4
C6—C1—H1	119.7	C8—C13—C12	121.3 (2)
C2—C1—H1	119.7	C8—C13—H13	119.3
C3—C2—C1	120.5 (2)	C12—C13—H13	119.3
C3—C2—H2	119.7	O1—C14—H14A	109.5
C1—C2—H2	119.7	O1—C14—H14B	109.5
C4—C3—C2	119.5 (2)	H14A—C14—H14B	109.5
C4—C3—H3	120.3	O1—C14—H14C	109.5
C2—C3—H3	120.3	H14A—C14—H14C	109.5
C3—C4—C5	120.3 (2)	H14B—C14—H14C	109.5
C3—C4—H4	119.9	C16—C15—C20	118.2 (2)
C5—C4—H4	119.9	C16—C15—S2	120.83 (19)
C4—C5—C6	120.7 (2)	C20—C15—S2	120.9 (2)
C4—C5—H5	119.6	C15—C16—C17	121.5 (3)
C6—C5—H5	119.6	C15—C16—H16	119.2
C1—C6—C5	118.41 (19)	C17—C16—H16	119.2
C1—C6—C7	119.62 (19)	C18—C17—C16	119.1 (3)
C5—C6—C7	121.96 (19)	C18—C17—H17	120.5
C6—C7—S1	109.22 (13)	C16—C17—H17	120.5
C6—C7—S2	113.90 (14)	C19—C18—O2	115.9 (3)
S1—C7—S2	107.73 (11)	C19—C18—C17	119.3 (3)
C6—C7—H7	108.6	O2—C18—C17	124.8 (3)
S1—C7—H7	108.6	C18—C19—C20	121.4 (3)
S2—C7—H7	108.6	C18—C19—H19	119.3
C9—C8—C13	118.7 (2)	C20—C19—H19	119.3
C9—C8—S1	117.84 (17)	C19—C20—C15	120.5 (3)
C13—C8—S1	123.28 (17)	C19—C20—H20	119.8
C10—C9—C8	120.6 (2)	C15—C20—H20	119.8
C10—C9—H9	119.7	O2—C21—H21A	109.5
C8—C9—H9	119.7	O2—C21—H21B	109.5
C9—C10—C11	120.6 (2)	H21A—C21—H21B	109.5
C9—C10—H10	119.7	O2—C21—H21C	109.5
C11—C10—H10	119.7	H21A—C21—H21C	109.5
O1—C11—C12	125.0 (2)	H21B—C21—H21C	109.5
O1—C11—C10	115.50 (19)	C11—O1—C14	118.12 (18)
C12—C11—C10	119.5 (2)	C18—O2—C21	118.2 (3)
C11—C12—C13	119.2 (2)	C8—S1—C7	102.63 (9)
C11—C12—H12	120.4	C15—S2—C7	100.37 (10)
			
C6—C1—C2—C3	−0.8 (4)	C20—C15—C16—C17	−1.4 (4)
C1—C2—C3—C4	0.8 (4)	S2—C15—C16—C17	−179.6 (2)
C2—C3—C4—C5	−0.2 (4)	C15—C16—C17—C18	2.0 (5)
C3—C4—C5—C6	−0.5 (4)	C16—C17—C18—C19	−0.9 (5)
C2—C1—C6—C5	0.1 (3)	C16—C17—C18—O2	178.1 (3)
C2—C1—C6—C7	178.7 (2)	O2—C18—C19—C20	−179.7 (3)
C4—C5—C6—C1	0.5 (3)	C17—C18—C19—C20	−0.6 (5)
C4—C5—C6—C7	−178.0 (2)	C18—C19—C20—C15	1.2 (5)
C1—C6—C7—S1	−129.59 (19)	C16—C15—C20—C19	−0.2 (4)
C5—C6—C7—S1	48.9 (2)	S2—C15—C20—C19	178.0 (2)
C1—C6—C7—S2	109.9 (2)	C12—C11—O1—C14	−1.1 (3)
C5—C6—C7—S2	−71.6 (2)	C10—C11—O1—C14	178.5 (2)
C13—C8—C9—C10	−1.1 (3)	C19—C18—O2—C21	−172.7 (3)
S1—C8—C9—C10	174.58 (16)	C17—C18—O2—C21	8.2 (5)
C8—C9—C10—C11	1.5 (3)	C9—C8—S1—C7	131.85 (17)
C9—C10—C11—O1	−179.77 (18)	C13—C8—S1—C7	−52.7 (2)
C9—C10—C11—C12	−0.2 (3)	C6—C7—S1—C8	168.12 (15)
O1—C11—C12—C13	178.0 (2)	S2—C7—S1—C8	−67.69 (13)
C10—C11—C12—C13	−1.5 (3)	C16—C15—S2—C7	−70.3 (2)
C9—C8—C13—C12	−0.6 (3)	C20—C15—S2—C7	111.6 (2)
S1—C8—C13—C12	−176.06 (17)	C6—C7—S2—C15	−73.83 (16)
C11—C12—C13—C8	1.9 (3)	S1—C7—S2—C15	164.86 (11)

### Hydrogen-bond geometry (Å, º)

Cg1 and Cg2 are the centroids of the C1–C6 and C8–C13 rings, respectively.

**Table:**

*D*—H···*A*	*D*—H	H···*A*	*D*···*A*	*D*—H···*A*
C4—H4···*Cg*2^i^	0.93	2.99	3.713 (3)	136
C10—H10···*Cg*2^ii^	0.93	2.96	3.610 (2)	128
C21—H21*B*···*Cg*1^iii^	0.96	2.98	3.914 (5)	165

Symmetry codes: (i) *x*, −*y*+3/2, *z*−1/2; (ii) −*x*, *y*+1/2, −*z*+1/2; (iii) −*x*+1, *y*−1/2, −*z*+1/2.
